# Association Between Osimertinib Dose Reduction and Treatment Outcomes in First‐Line EGFR‐Mutated Advanced NSCLC: The Impact of Post‐Progression Management

**DOI:** 10.1111/1759-7714.70328

**Published:** 2026-06-17

**Authors:** Issei Oi, Kohei Fujita, Takuma Imakita, Mitsuhiro Tukino, Tetsuo Noguchi, Yuta Okada, Saiki Yoshimura, Naoki Fujimoto, Shogo Toyama, Atsuko Watanabe, Takanori Ito, Ryosuke Kaku, Osamu Kanai, Masatsugu Ohuchi, Kiminobu Tanizawa, Satoru Sawai, Tadashi Mio

**Affiliations:** ^1^ Division of Respiratory Medicine, Center for Respiratory Diseases National Hospital Organization Kyoto Medical Center Kyoto Kyoto Japan; ^2^ Department of Respiratory Medicine Hikone Municipal Hospital Hikone Shiga Japan; ^3^ Department of Respiratory Medicine and Thoracic Surgery Nagahama City Hospital Nagahama Shiga Japan; ^4^ Department of Thoracic Surgery, Center for Respiratory Medicine National Hospital Organization Kyoto Medical Center Kyoto Kyoto Japan

**Keywords:** best supportive care, dose reduction, EGFR mutation, non‐small cell lung cancer, osimertinib, overall survival, time to treatment failure

## Abstract

**Background:**

Osimertinib is the standard first‐line treatment for epidermal growth factor receptor (EGFR)‐mutated advanced non‐small cell lung cancer (NSCLC). However, the impact of dose‐reduction on survival and the role of post‐progression management in real‐world settings remain unclear.

**Methods:**

We conducted a multi‐institutional retrospective cohort study on patients with EGFR‐mutated advanced NSCLC who received first‐line osimertinib between January 2018 and December 2023. Patients were classified into dose‐reduced and full‐dose groups. Primary endpoints were time to treatment failure (TTF) and overall survival (OS). The subsequent treatment after progression was also exploratively assessed to evaluate the impact of initial dose modification on the entire treatment sequence.

**Results:**

A total of 90 patients were analyzed, consisting of 61 patients in the full‐dose group and 29 in the dose‐reduction group. Median follow‐up was 21.5 months (IQR 9.1–37.2). The dose‐reduced group had significantly worse baseline performance status (PS) (PS 2–3: 37.9% vs. 14.8%, *p* = 0.014). Despite this, dose reduction showed no significant impact on TTF (adjusted HR 0.66, 95% CI 0.40–1.10, *p* = 0.11) or OS (HR 0.97, 95% CI 0.59–1.58, *p* = 0.89). Multivariate analysis revealed that transition to best supportive care (BSC) after progression, rather than initial dose reduction, was associated with OS (hazard ratio, 7.40; *p* < 0.0001). The proportions of patients receiving subsequent chemotherapy were comparable between the groups.

**Conclusions:**

Osimertinib dose reduction in the first‐line setting was not associated with inferior OS, suggesting that appropriate dose modification helps maintain PS. Transition to BSC after progression, rather than initial dose intensity, was associated with poor prognosis.

## Graphical abstract

In first‐line EGFR‐mutated advanced NSCLC, osimertinib dose reduction was not associated with worse overall survival. Appropriate dose modification may preserve patient fitness and improve access to subsequent therapy, highlighting the importance of post‐progression management.

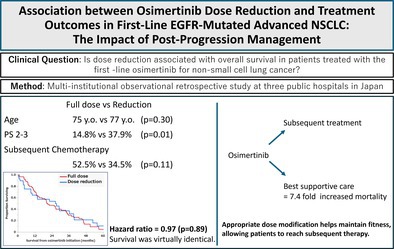

## 1 Introduction

Lung cancer remains the leading cause of cancer‐related mortality worldwide, with non‐small cell lung cancer (NSCLC) accounting for approximately 85% of cases [1]. Epidermal growth factor receptor (EGFR) mutations, particularly exon 19 deletions and exon 21 L858R point mutations, occur in 10%–15% of Caucasian patients and 40%–50% of Asian patients with lung adenocarcinoma [2, 3]. These mutations serve as actionable targets for tyrosine kinase inhibitors (TKIs).

Osimertinib, a third‐generation irreversible EGFR‐TKI, has revolutionized the treatment landscape for EGFR‐mutated advanced NSCLC. The pivotal FLAURA trial demonstrated superior progression‐free survival (PFS) and overall survival (OS) compared to first‐generation EGFR‐TKIs (gefitinib or erlotinib) in treatment‐naïve patients, establishing osimertinib as the standard first‐line therapy [4, 5]. The median PFS was 18.9 months with osimertinib versus 10.2 months with comparator EGFR‐TKIs, and the median OS was 38.6 months versus 31.8 months [5].

Despite its efficacy, osimertinib is associated with adverse events (AEs) that occasionally necessitate dose reduction. Common AEs include dermatologic toxicities (rash, paronychia, dry skin), gastrointestinal symptoms (diarrhea, anorexia), interstitial lung disease (ILD), and cardiac toxicities [4, 5]. In clinical trials, dose reduction was performed in 5% of patients [4], and reports indicate that in actual clinical practice, dose reduction occurs at a higher rate of 15%–30% [6, 7, 8].

The clinical implications of osimertinib dose reduction remain incompletely understood. A key unresolved issue is how dose reduction influences treatment continuity and subsequent management after disease progression. Most prior studies have focused primarily on comparisons of survival outcomes such as PFS or OS, without fully capturing treatment discontinuation dynamics or the downstream therapeutic trajectory [6, 7, 8, 9]. In the multicenter prospective observational study by Awano et al., a significant prolongation of PFS was observed in the weight reduction group compared to the standard‐dose group (HR 0.67, *p* < 0.001), but no significant difference was observed in OS (HR 0.82, *p* = 0.15) [7]. In contrast, a single‐center retrospective study in the United States by Barsouk et al. showed a significant shortening of PFS in the reduced‐dose group (17.0 vs. 24.6 months, *p* = 0.043), but no difference in OS (36.7 vs. 39.2 months, *p* = 0.749) [8]. In routine clinical practice, access to active post‐progression therapy—or conversely, transition to best supportive care (BSC)—may substantially influence patient prognosis [10]. However, the relationship between dose reduction and these post‐treatment pathways has not been clearly defined.

This issue is becoming increasingly relevant in aging populations and in patients with comorbidities, in whom treatment tolerability frequently necessitates dose modification. Therefore, in the present study, we investigated the impact of osimertinib dose reduction on time to treatment failure (TTF) and OS in patients receiving first‐line therapy for *EGFR* mutation‐positive advanced NSCLC, with particular attention to the role of post‐progression management strategies, including the receipt of further active treatment versus transition to BSC. Through this approach, we aimed to reassess the clinical significance of dose modification within the context of the entire treatment course.

## 2 Materials and Methods

### 2.1 Study Design and Patient Selection

This multi‐institutional retrospective cohort study was conducted at three Japanese public hospitals. The Kyoto Medical Center is a 600‐bed facility in Kyoto, Kyoto; Hikone Municipal Hospital has 438 beds in Hikone, Shiga; and Nagahama Municipal Hospital has 565 beds in Nagahama, Shiga, all of which serve as public hospitals. This study was conducted in accordance with the Declaration of Helsinki and approved by the Ethical Review Committee of Kyoto Medical Center (order number 23‐022) and the relevant committees of each institution. Because of the retrospective nature of the study and the absence of patient‐identifiable data, written informed consent was not required. Instead, informed consent was obtained through an opt‐out procedure in accordance with local ethical guidelines.

Eligible patients met the following inclusion criteria: (1) histologically or cytologically confirmed advanced or recurrent NSCLC harboring activating *EGFR* mutation; (2) received first‐line osimertinib treatment between January 2018 and December 2023; (3) age ≥ 18 years; and (4) adequate organ function to initiate osimertinib therapy.

Exclusion criteria included: (1) concurrent malignancies requiring active treatment; (2) insufficient medical records to determine treatment outcomes; (3) osimertinib administered as second‐line or later therapy; (4) before disease progression or treatment failure; and (5) postoperative osimertinib, concurrent chemotherapy, and attending clinical trial.

### 2.2 Data Collection

Comprehensive clinical data were extracted from electronic medical records, including patient demographics (age, sex), Eastern Cooperative Oncology Group (ECOG) performance status (PS), *EGFR* mutation type (major mutations: exon 19 deletion or exon 21 L858R vs. other mutations), AE profiles, and subsequent treatment strategies.

Dose reduction was defined as any decrease from the standard starting dose of 80 mg daily that was sustained for ≥ 2 consecutive weeks. Dose modifications were performed at the treating physician's discretion following shared decision‐making with patients, based on AE severity, tolerability, PS, comorbidities, and patient preference. Permitted dose reductions included: 80 mg every other day, 40 mg once daily, or 40 mg every other day. Patients were classified as dose‐reduced if they received any sustained dose lower than 80 mg daily, or full‐dose if they maintained 80 mg daily throughout treatment.

Transition to BSC was defined as the clinical decision to forgo further systemic anticancer therapy following osimertinib discontinuation. This decision was made through informed consent discussions between patients, families, and physicians, considering disease status, PS, comorbidities, prior toxicities, patient preferences, and anticipated quality of life. BSC included symptom‐directed palliative interventions but excluded systemic therapies (chemotherapy, targeted agents, immunotherapy). Patients receiving any systemic anticancer therapy after osimertinib were classified as receiving “active subsequent therapy.” Because this was a retrospective study, AE severity was not systematically graded using Common Terminology Criteria for Adverse Events (CTCAE), but AE types were documented and categorized.

### 2.3 Study Endpoints

The co‐primary endpoints were TTF and OS. TTF was defined as the time from osimertinib initiation until treatment failure for any reason (disease progression, AEs, patient preference, or death). OS was defined as the time from osimertinib initiation until death from any cause. Patients alive at the time of analysis were censored at the date of last follow‐up. Secondary endpoints included identification of prognostic factors for TTF and OS.

### 2.4 Statistical Analysis

Continuous variables were summarized as medians with interquartile ranges (IQR) and compared using the Mann–Whitney *U* test. Categorical variables were expressed as frequencies and percentages and compared using chi‐square or Fisher's exact test.

Survival curves were generated using the Kaplan–Meier method and compared using the log‐rank test. Univariate Cox proportional hazards regression identified potential prognostic factors for TTF and OS. For multivariate analysis of TTF, variables with *p* < 0.15 in univariate analysis were included. For OS, variables with *p* < 0.10 were included. To minimize the impact of immortal time bias, a landmark analysis was performed by including only patients who remained on treatment and were alive at 60 days after the initiation of osimertinib.

The association between baseline PS and subsequent BSC transition was assessed using Fisher's exact test and odds ratios with 95% confidence intervals. Due to observed collinearity between baseline ECOG PS and post‐progression BSC transition (OR 6.34, *p* < 0.001), sensitivity analyses used alternative models: Model 1 (PS‐adjusted, excluding BSC), Model 2 (including post‐progression BSC transition as an exploratory covariate), and Model 3 (full model including both variables with variance inflation factors calculated). Hazard ratios with 95% confidence intervals were calculated. *p* < 0.05 (two‐sided) was considered significant.

All analyses used JMP Student Edition 18 (SAS Institute Inc., Cary, NC, USA).

## 3 Results

### 3.1 Patient Characteristics

**FIGURE 1:**
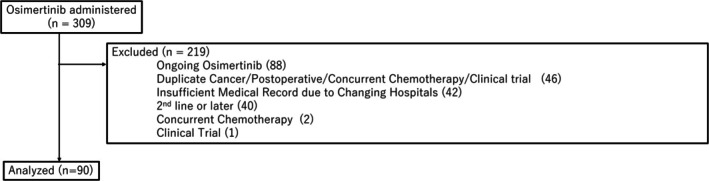
Patient flow diagram. Osimertinib was administered to 309 patients. A total of 219 patients were excluded, and finally 90 patients were analyzed in this study.

During the study period, osimertinib was administered to 309 patients. A total of 219 patients were excluded, and finally a total of 90 patients with EGFR‐mutated advanced NSCLC who received first‐line osimertinib were included in this analysis (Figure 1). The median follow‐up duration was 21.5 months (IQR 9.1–37.2 months) for the entire cohort. Twenty‐nine patients (32.2%) underwent dose reduction during treatment (dose‐reduced group), while 61 patients (67.8%) received full dose (full‐dose group). The specific dose‐reduction regimens were as follows: 27 patients (93.1%) received 40 mg daily, one patient received 40 mg every other day, and one patient received 80 mg every other day.

Baseline patient characteristics are summarized in Table 1. The median age was 75 years (IQR 70.5–80.0) in the full‐dose group and 77 years (IQR 70.0–83.5) in the dose‐reduced group (*p* = 0.30). The dose‐reduced group had a lower proportion of male patients (27.6% vs. 49.2%), although this difference did not reach statistical significance (*p* = 0.053). Notably, patients who underwent dose reduction had significantly worse baseline ECOG PS, with 37.9% having PS 2–3 compared to 14.8% in the full‐dose group (*p* = 0.014). The distribution of EGFR mutation types was balanced between groups, with major mutations present in 93.1% of dose‐reduced patients and 91.8% of full‐dose patients (*p* = 0.83).

**TABLE 1:** Patient characteristics.

	Full‐dose (*n* = 61)	Dose‐reduced (*n* = 29)	*p*
Age, median (IQR) (years)	75 (70.5–80.0)	77 (70.0–83.5)	0.30
Male sex, *n* (%)	30 (49.2)	8 (27.6)	0.053
ECOG PS 2–3, *n* (%)	9 (14.8)	11 (37.9)	0.014
Major mutations, *n* (%)	56 (91.8)	27 (93.1)	0.83
Any adverse events, *n* (%)	36 (59.0)	21 (72.4)	0.22
Dermatologic toxicity, *n* (%)	16 (26.2)	7 (24.1)	0.83
Interstitial lung disease, *n* (%)	11 (18.0)	3 (10.3)	0.35
Liver dysfunction, *n* (%)	0 (0)	4 (13.8)	0.02
Cardiac arrhythmia, *n* (%)	0 (0)	4 (13.8)	0.02
Malaise/anorexia, *n* (%)	18 (29.5)	12 (41.4)	0.26
Post‐progression BSC, *n* (%)	7 (11.5)	10 (34.5)	0.15
Cytotoxic chemotherapy after osimertinib	32 (52.5)	10 (34.5)	0.11
Platinum‐based chemotherapy after osimertinib, *n* (%)	28 (45.9)	9 (31.0)	0.18
Continued osimertinib after progression, *n* (%)	9 (14.8)	5 (17.2)	0.71
Immuno‐oncology monotherapy, *n* (%)	3 (4.9)	0	0.27

Abbreviations: BSC, best supportive care; ECOG PS, Eastern Cooperative Oncology Group performance status; IQR, interquartile range.

While the overall incidence of any AE was numerically higher in the dose‐reduced group (72.4% vs. 59.0%), this difference was not statistically significant (*p* = 0.22). Specific AE patterns differed between groups: hepatic enzyme elevation and cardiac arrhythmias were observed exclusively in the dose‐reduced group (13.8%) each. Dermatologic toxicities and fatigue/anorexia were similarly distributed between groups.

Importantly, the proportion of patients who transitioned to BSC following osimertinib discontinuation was numerically higher in the dose‐reduced group (34.5% vs. 11.5%, *p* = 0.15), although this difference did not reach statistical significance. This trend likely reflects the poorer baseline functional status and reduced tolerance for subsequent therapies in patients requiring dose reduction. Among patients who progressed on osimertinib, the proportions receiving subsequent cytotoxic chemotherapy (34.5% in the reduction group vs. 52.5% in the full‐dose group) and continued osimertinib treatment (17.2% vs. 14.8%) were comparable (Table 1).

### 3.2 Dose Reduction and TTF

**FIGURE 2:**
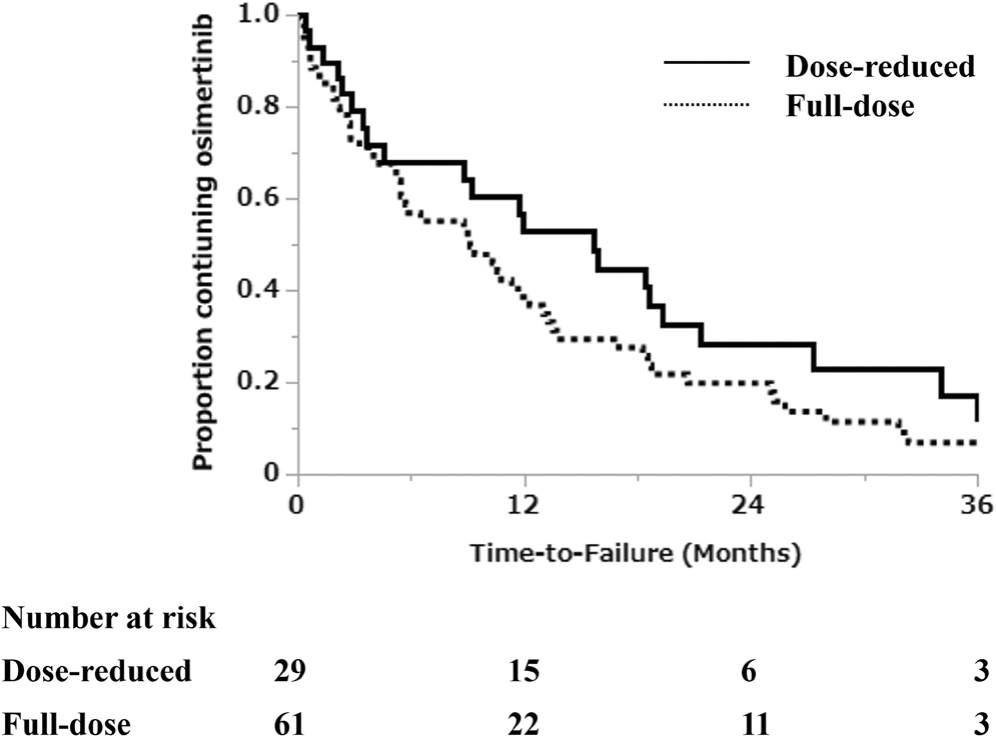
Time to treatment failure by osimertinib dose. Kaplan–Meier curves for time to treatment failure comparing dose‐reduced (*n* = 29) versus full‐dose (*n* = 61) osimertinib groups. Dose reduction showed a trend toward longer TTF (HR 0.66, 95% CI 0.40–1.10, *p* = 0.11). Shaded areas represent 95% CI. Number at risk shown below the graph.

There were 90 events in total (26 in the dose‐reduction group and 64 in the full‐dose group). The median TTF was numerically longer in the dose‐reduced group compared to the full‐dose group, although this difference did not reach statistical significance in unadjusted analysis (Figure 2). The unadjusted hazard ratio for treatment failure with dose reduction was 0.66 (95% CI 0.40–1.10, *p* = 0.11), suggesting a trend toward prolonged treatment duration with dose reduction.

Univariate Cox regression analysis identified several factors associated with TTF (Table 2). Major mutations were associated with significantly longer TTF compared to minor mutations (HR 0.41, 95% CI 0.18–0.97, *p* = 0.04). Presence of ILD was significantly associated with shorter TTF (HR 2.20, 95% CI 1.16–4.14, *p* = 0.02). Dose reduction showed a trend toward longer TTF (HR 0.66, 95% CI 0.40–1.10, *p* = 0.11), while age, sex, PS, and other AEs were not significantly associated with TTF.

**TABLE 2:** Univariate and multivariate Cox regression analysis for time to treatment failure.

	Univariate analysis		Multivariate model	
	HR (95% CI)	*p*	aHR (95% CI)	*p*
Age	1.01 (0.98–1.04)	0.51	—	—
Male sex	1.20 (0.76–1.91)	0.44	—	—
ECOG PS 2–3	1.34 (0.79–2.28)	0.28	—	—
Major mutation	0.41 (0.18–0.97)	0.04	0.47 (0.20–1.12)	0.09
Dermatologic toxicity	0.94 (0.53–1.67)	0.84		
Interstitial lung disease	2.20 (1.16–4.14)	0.02	2.14 (1.13–4.05)	0.02
Liver dysfunction	0.66 (0.16–2.70)	0.56		
Cardiac arrhythmia	0.54 (0.20–1.49)	0.24		
Malaise/anorexia	1.21 (0.74–1.96)	0.45		
Dose reduction	0.66 (0.40–1.10)	0.11	0.70 (0.42–1.17)	0.18

Abbreviations: aHR, adjusted hazard ratio; BSC, best supportive care; CI, confidence interval; ECOG PS, Eastern Cooperative Oncology Group performance status; HR, hazard ratio.

In multivariate analysis including major mutations, ILD, and dose reduction (variables with *p* < 0.15 in univariate analysis), dose reduction did not reach statistical significance (adjusted HR 0.70, 95% CI 0.42–1.17, *p* = 0.18). However, ILD remained a significant predictor of shorter TTF (adjusted HR 2.14, 95% CI 1.13–4.05, *p* = 0.019), while major mutations showed a trend toward longer TTF (adjusted HR 0.47, 95% CI 0.20–1.12, *p* = 0.089).

### 3.3 Dose Reduction and OS

**FIGURE 3:**
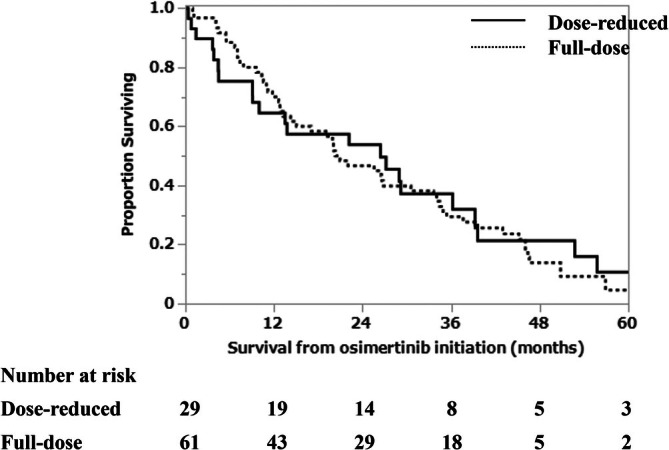
Overall survival by osimertinib dose. Kaplan–Meier curves for overall survival comparing dose‐reduced (*n* = 29) versus full‐dose (*n* = 61) osimertinib groups. OS was virtually identical (HR 0.97, 95% CI 0.59–1.58, *p* = 0.89), despite the trend toward longer TTF in the dose‐reduced group (Figure 2). Number at risk shown below the graph.

There were 76 events in total (24 in the dose‐reduction group and 52 in the full‐dose group). Despite the trend toward longer TTF observed with dose reduction, OS was similar between the dose‐reduced and full‐dose groups (Figure 3). The unadjusted hazard ratio for death with dose reduction was 0.97 (95% CI 0.59–1.58, *p* = 0.89), indicating virtually identical survival outcomes regardless of dose strategy. In a landmark analysis excluding patients with early death or treatment discontinuation within 60 days (*n* = 74), the association between dose reduction and OS remained non‐significant (HR 0.90, 95% CI 0.53–1.53, *p* = 0.69, Figure S1).

Univariate Cox regression for OS (Table 3) revealed that poor baseline ECOG PS (2–3) was associated with worse survival (HR 1.72, 95% CI 1.02–2.92, *p* = 0.04), and major mutations showed a strong trend toward improved survival (HR 0.44, 95% CI 0.19–1.02, *p* = 0.055). Dermatologic toxicity showed a trend toward improved survival (HR 0.60, 95% CI 0.35–1.05, *p* = 0.072). Most strikingly, transition to BSC was by far the most powerful predictor of mortality (HR 7.40, 95% CI 3.93–13.93, *p* < 0.0001). Dose reduction showed no association with OS in univariate analysis (HR 0.97, *p* = 0.89).

**TABLE 3:** Univariate and multivariate Cox regression analysis for overall survival.

	Univariate analysis		Multivariate Model 1		Multivariate Model 2		Multivariate Model 3	
	HR	*p*	aHR (95% CI)	*p*	aHR (95% CI)	*p*	aHR (95% CI)	*p*
Age	1.01 (0.99–1.05)	0.35						
Male sex	1.25 (0.80–1.98)	0.33						
ECOG PS 2–3	1.72 (1.02–2.92)	0.04	1.72 (1.02–2.90)	0.04			1.08 (0.60–1.93)	0.80
Major mutation	0.44 (0.19–1.02)	0.06	0.46 (0.19–1.07)	0.07	0.41 (0.17–0.97)	0.04	0.42 (0.18–0.99)	0.047
Dermatologic toxicity	0.60 (0.35–1.05)	0.07	0.77 (0.41–1.27)	0.26	0.72 (0.62–1.98)	0.72	0.85 (0.48–1.50)	0.57
Interstitial lung disease	1.10 (0.64–1.89)	0.72						
Liver dysfunction	1.29 (0.40–4.14)	0.67						
Cardiac arrhythmia	0.59 (0.21–1.63)	0.31						
Malaise/anorexia	1.08 (0.67–1.74)	0.74						
Post‐progression BSC	7.40 (3.93–13.93)	< 0.0001			3.82 (2.08–7.02)	< 0.0001	7.07 (3.48–14.38)	< 0.0001
Dose reduction	0.97 (0.59–1.58)	0.89						

Abbreviations: aHR, adjusted hazard ratio; BSC, best supportive care; CI, confidence interval; ECOG PS, Eastern Cooperative Oncology Group performance status; HR, hazard ratio.

Three multivariate models were constructed (Table 3). Variables with *p* < 0.10 in univariate analysis (PS, major mutations, dermatologic toxicity, and BSC) were candidates for inclusion, with different combinations tested across models to address collinearity. In Model 1 (PS‐adjusted, excluding BSC), PS 2–3 remained a significant predictor of mortality (aHR 1.72, 95% CI 1.02–2.90, *p* = 0.043), while major mutations showed a trend toward improved survival (aHR 0.46, *p* = 0.072). In Model 2 (including post‐progression BSC transition as an exploratory covariate), major mutations became a significant independent predictor of improved survival (aHR 0.41, 95% CI 0.17–0.97, *p* = 0.041), while dermatologic toxicity did not retain significance (aHR 0.72, *p* = 0.718). Most importantly, BSC transition emerged as the dominant prognostic factor with a nearly 4‐fold increased risk of death compared to active subsequent therapy (aHR 3.82, 95% CI 2.08–7.02, *p* < 0.0001). In Model 3 (full model including both PS and BSC), the PS effect was completely attenuated and lost statistical significance (aHR 1.08, 95% CI 0.60–1.93, *p* = 0.802), while BSC remained highly significant (aHR 7.07, 95% CI 3.48–14.38, *p* < 0.0001). However, the substantially wider confidence interval for BSC in Model 3 compared to Model 2 suggests estimation instability due to collinearity between PS and BSC. Major mutations remained a significant protective factor across all models (aHR 0.41–0.46).

## 4 Discussion

In this multi‐institutional retrospective study, first‐line osimertinib dose reduction was not associated with inferior OS compared with the full‐dose treatment. Our findings suggest that OS may be influenced more by post‐progression management than by initial dose intensity. The comparable proportions of patients receiving subsequent chemotherapy or continued osimertinib in the two groups support the hypothesis that appropriate dose modification for toxicity may help preserve PS and maintain access to later active treatment. Given the retrospective design, these findings should be interpreted as exploratory; however, they suggest that treatment tolerability and the post‐progression care pathway are important considerations when evaluating the clinical impact of dose reduction in EGFR‐mutated advanced NSCLC.

Previous real‐world studies of first‐line osimertinib dose reduction have reported conflicting findings for progression‐related outcomes. Some studies suggested longer PFS in dose‐reduced patients [7], whereas others reported shorter progression‐related outcomes compared with full‐dose treatment. Notably, however, OS was broadly similar across these reports [8]. Our findings suggest that this discrepancy may partly reflect differences in post‐progression management rather than the effect of initial dose intensity alone. In particular, transition to BSC, as opposed to receipt of active subsequent therapy, may have a substantial influence on long‐term survival. This interpretation may help explain why progression‐related endpoints have varied across studies whereas OS has been less clearly affected by dose reduction.

Our sensitivity analyses further support this interpretation by showing that the relationship between baseline PS and survival is closely linked to post‐progression management. Because poor baseline PS was strongly associated with subsequent transition to BSC, the prognostic impact initially attributed to PS may partly reflect limited access to active subsequent therapy. At the same time, the presence of collinearity and the post‐baseline nature of BSC require cautious interpretation of these findings. Taken together, these results should be regarded as exploratory, but they support the view that long‐term outcomes may depend more on the continuity of care after progression than on initial dose intensity alone. The observed survival outcomes despite dose reduction may be supported by the pharmacologic profile of osimertinib. Osimertinib has a 48‐h half‐life and accumulates to steady‐state over weeks [11]. The AURA Phase I study demonstrated comparable response rates between 40 (43%) and 80 mg (52%) cohorts [12], and preclinical studies showed that 40 mg daily achieves plasma concentrations exceeding the IC_90_ for EGFR mutations [13], suggesting that our dose‐reduced patients who would have lower body weight may achieve therapeutic levels at reduced doses.

Our findings have important practical implications. First, dose reduction may be considered for AE management without clear evidence of inferior survival, particularly in elderly or frail populations. Second, maintaining eligibility for subsequent therapy throughout the disease course may be clinically important. The strong association between BSC transition and mortality suggests that access to active post‐progression therapy substantially influences long‐term outcomes. Clinicians should therefore consider feasible post‐progression treatment strategies whenever appropriate [10, 14, 15, 16, 17, 18].

Several limitations warrant acknowledgment. First, the retrospective design and relatively small sample size (*n* = 90) may limit statistical power and generalizability. Larger, prospective multi‐center studies are warranted to further elucidate the clinical significance of osimertinib dose modification. Additionally, we could not provide a detailed breakdown of the exact cumulative dose or the specific toxicity according to the CTCAE, for all patients due to the retrospective nature of the medical record review. Second, the Japanese population may have different pharmacokinetics than Western populations [19, 20], potentially limiting generalizability. Third, the assessment of BSC transition was based on clinical records, and we could not fully account for patient preferences, family circumstances, or socioeconomic factors that may have influenced the decision to forgo subsequent active therapy. The reasons for BSC transition (patient refusal, physician decision, poor PS, rapid deterioration) were not systematically categorized, which may represent heterogeneous clinical scenarios. Fourth, although we performed a landmark analysis to reduce the risk of immortal time bias, bias related to the definition and timing of dose reduction cannot be fully excluded. Finally, patient‐reported outcomes were unavailable, precluding assessment of whether dose reduction improved quality of life from the patient's perspective [21, 22]. Therefore, the results regarding the impact of dose reduction should be interpreted as exploratory and hypothesis‐generating.

## 5 Conclusions

Osimertinib dose reduction was not associated with inferior OS in first‐line EGFR‐mutated advanced NSCLC. Survival appeared to be more strongly influenced by access to active post‐progression therapy than by initial dose intensity. These findings suggest that maintaining treatment continuity throughout the disease course may be clinically important.

## Author Contributions

**Mitsuhiro Tukino:** methodology, data curation, investigation. **Issei Oi:** conceptualization, methodology, data curation, investigation, formal analysis, supervision, visualization, writing – original draft, project administration. **Kohei Fujita:** conceptualization, methodology, funding acquisition, writing – review and editing. **Tetsuo Noguchi:** methodology, investigation, data curation. **Takuma Imakita:** conceptualization, methodology. **Naoki Fujimoto:** data curation, investigation, project administration. **Ryosuke Kaku:** data curation, investigation. **Atsuko Watanabe:** investigation, data curation. **Saiki Yoshimura:** data curation, investigation, project administration. **Osamu Kanai:** methodology, data curation, investigation, formal analysis. **Satoru Sawai:** investigation, data curation, methodology. **Takanori Ito:** data curation, investigation. **Yuta Okada:** data curation, investigation. **Masatsugu Ohuchi:** investigation, data curation. **Kiminobu Tanizawa:** methodology, conceptualization, supervision. **Shogo Toyama:** investigation, data curation. **Tadashi Mio:** methodology, supervision, conceptualization.

## Funding

The authors have nothing to report.

## Conflicts of Interest

The authors declare no conflicts of interest.

## Supporting information


**Figure S1:** Kaplan–Meier curves for overall survival in the landmark analysis excluding patients with early death or treatment discontinuation within 60 days.

## Data Availability

The data that support the findings of this study are available from the corresponding author upon reasonable request.
