# Supplementary figures and images for: Association Between Osimertinib Dose Reduction and Treatment Outcomes in First‐Line EGFR‐Mutated Advanced NSCLC: The Impact of Post‐Progression Management
Source: Thorac Cancer. 2026 Jun 17;17(12):e70328. doi: 10.1111/1759-7714.70328 (PMC13275180; doi:10.1111/1759-7714.70328)

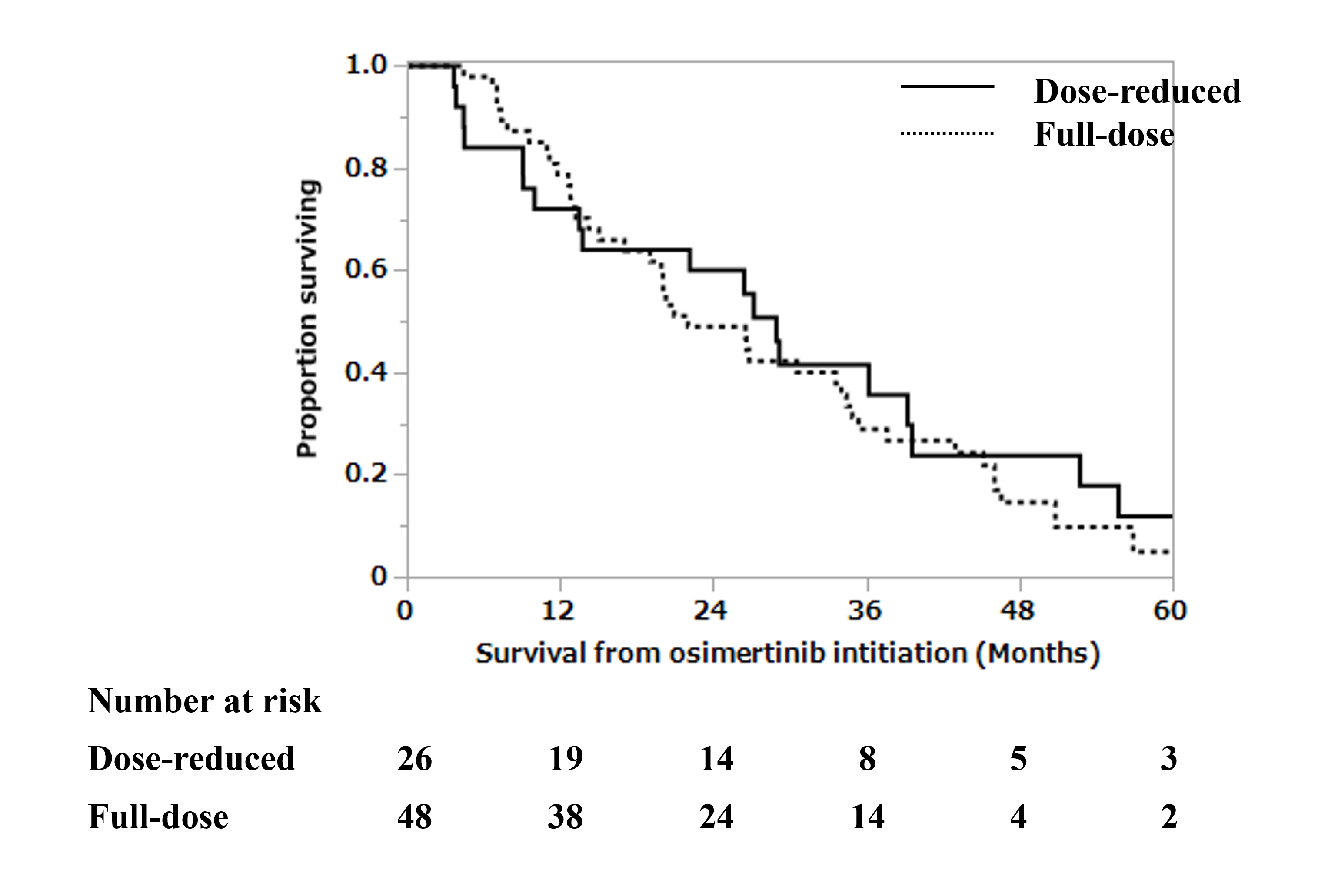

Supplement: Supplementary file 1 — Figure S1: Kaplan–Meier curves for overall survival in the landmark analysis excluding patients with early death or treatment discontinuation within 60 days. [file TCA-17-e70328-s001.png]
